# Recent Advances, Approaches and Challenges in the Development of Universal Influenza Vaccines

**DOI:** 10.1111/irv.13276

**Published:** 2024-03-21

**Authors:** Caryn Myn Li Lim, Thamil Vaani Komarasamy, Nur Amelia Azreen Binti Adnan, Ammu Kutty Radhakrishnan, Vinod R. M. T. Balasubramaniam

**Affiliations:** ^1^ Infection and Immunity Research Strength, Jeffrey Cheah School of Medicine & Health Sciences Monash University Malaysia Bandar Sunway Malaysia

**Keywords:** haemagglutinin, influenza virus, neutralizing antibodies, universal influenza vaccine

## Abstract

Every year, influenza virus infections cause significant morbidity and mortality worldwide. They pose a substantial burden of disease, in terms of not only health but also the economy. Owing to the ability of influenza viruses to continuously evolve, annual seasonal influenza vaccines are necessary as a prophylaxis. However, current influenza vaccines against seasonal strains have limited effectiveness and require yearly reformulation due to the virus undergoing antigenic drift or shift. Vaccine mismatches are common, conferring suboptimal protection against seasonal outbreaks, and the threat of the next pandemic continues to loom. Therefore, there is a great need to develop a universal influenza vaccine (UIV) capable of providing broad and durable protection against all influenza virus strains. In the quest to develop a UIV that would obviate the need for annual vaccination and formulation, a multitude of strategies is currently underway. Promising approaches include targeting the highly conserved epitopes of haemagglutinin (HA), neuraminidase (NA), M2 extracellular domain (M2e) and internal proteins of the influenza virus. The identification and characterization of broadly neutralizing antibodies (bnAbs) targeting conserved regions of the viral HA protein, in particular, have provided important insight into novel vaccine designs and platforms. This review discusses universal vaccine approaches presently under development, with an emphasis on those targeting the highly conserved stalk of the HA protein, recent technological advancements used and the future prospects of a UIV in terms of its advantages, developmental obstacles and potential shortcomings.

## 1 Introduction

Influenza is an acute respiratory disease that is highly contagious. Its manifestations range from mild to severe, characterized by a variety of symptoms [1]. Among the common presenting symptoms of influenza, this includes fever, sore throat, cough, chills, nasal discharge, malaise and myalgias. Illnesses such as pneumonia and bronchitis can develop as a result of severe or lethal cases of influenza, leading to death [2]. The influenza virus consists of four types: types A, B, C and D, two of which (influenza A and B viruses) cause seasonal epidemics in humans [1]. Influenza Type A displays a greater propensity for mutation, resulting in a heightened level of diversity in both its antigenic properties and virulence when compared to other influenza types. As influenza A viruses (IAVs) also circulate in animals, the zoonotic origin strains of IAV have the ability to cause sporadic pandemics [3, 4].

Influenza viruses are enveloped, segmented, negative‐sense single‐stranded RNA viruses belonging to the family *Orthomyxoviridae*. Both IAVs and influenza B viruses (IBVs) possess eight RNA segments, which encode numerous proteins including RNA polymerase subunits, viral surface glycoproteins haemagglutinin (HA), neuraminidase (NA), NP, matrix protein (M1), membrane protein (M2), the non‐structural protein NS1 and nuclear export protein (NEP) [5]. HA is found most abundantly on the viral surface and plays an essential role in the entry of the virus into host cells due to the function of its globular head and stem regions in mediating the binding to sialyloligosaccharide virus receptors and fusion with the membrane of host cells. The sialidase activity of NA facilitates the release of viral particles from the surface of host cells by cleaving off the sialic acid. The surface glycoproteins HA and NA of IAVs classify them into subtypes: 18 HA subtypes (H1–18) and 11 NA subtypes (N1–11). While IAVs have the capability to infect a broad range of avian and mammalian species, it is important to note that only three subtypes, namely, H1N1, H2N2 and H3N2, are known for their efficient infection and transmission among humans. H3N2 is indeed widely recognized as one of the predominant subtypes of IAV that has been circulating in the human population since the Hong Kong flu pandemic of 1968 [6].

Every year, seasonal epidemics of IAVs and IBVs cause a major disease burden in humans. In temperate regions (December to April in the Northern Hemisphere, June to September in the Southern Hemisphere), influenza outbreaks typically occur during the colder months, owing to temperature ambient conditions low in humidity [7]. In contrast, influenza epidemics in tropical regions are unpredictable, with infections occurring throughout the year. Globally, 290,000 to 650,000 deaths and 3–5 million severe influenza cases have been estimated to occur as a consequence of these annual epidemics, as reported by the World Health Organization (WHO) [8]. Currently, the H1N1pdm09 and H3N2 subtypes of IAV and the Yamagata and Victorian IBV lineages are the prevalent influenza viruses causing seasonal epidemics in humans [9].

In the past 100 years, there have been four influenza pandemics incurring high mortality rates involving millions worldwide. The Spanish influenza virus A (H1N1) that began in 1918 was the first and most devastating pandemic recorded, killing 50–100 million people in 1918–1919. In 1957, the second recorded pandemic caused by H2N2 (Asian influenza virus A) resulted in 1.1 million deaths from 1957 to 1959 [9]. The H3N2 Hong Kong influenza pandemic was associated with over million deaths globally. The most recent pandemic, the H1N1 swine influenza, caused millions of infections in over 200 countries and thousands of deaths [10].

The nature of the influenza virus as a constantly evolving pathogenic threat to humans via antigenic changes is a persistent problem that significantly interferes with current vaccination efforts. The two mechanisms altering the genetic composition of the virus are the antigenic drift and shift. The antigenic drift phenomenon involves minor changes occurring during replication in the genes encoding antigens, which result in alterations in the way they appear to the immune system [11]. While the antigenic shift, in which the segmental arrangement of the virus genome enables the reassortment of segments from different strains, leading to segmental exchange, may result in the periodic emergence of novel influenza strains. These pandemic strains have increased pathogenic ability in infecting humans during outbreaks [12].

An important lesson from previous influenza pandemics is that it is impossible to predict the specific subtype that will cause the outbreak. Besides the four pandemics, there has been an increase in the number of human infections and deaths from zoonotic influenza viruses such as avian (H5N1, H7N9 and H9N2) and swine (H1N1, H1N2 and H3N2) in recent years compared to decades previously [13]. Although the transmission of these ‘pre pandemic influenza viruses’ is currently limited to zoonosis thus far, there is concern regarding the ability of the virus to undergo viral shifts and drifts permitting human transmission and eventually causing a new influenza pandemic [13, 14].

Annual strain‐specific seasonal influenza vaccines are the cornerstone in preventing and reducing the burden of both seasonal and pandemic influenza. Through epidemics, the evolutionary nature of the viruses and surveillance in viral genome changes are traced by a tracking system led by the WHO, which then chooses the most suitable antigens for their inclusion in the development of seasonal influenza vaccines [11]. There is a significant challenge in developing commercial vaccines that provide sufficient breadth and depth of protection. The range of seasonal influenza vaccine effectiveness is 10%–60% [15]. In instances where mismatches occur between vaccine strains and circulating strains, vaccine effectiveness drops to very low values [14]. Factors such as egg‐based manufacturing further contribute to the virus acquiring mutations. In addition, host factors such as immunization history, previous influenza exposure, age and comorbidities may lead to suboptimal vaccine effectiveness [16].

## 2 Pathogenesis of Influenza Virus

In the pathogenicity of IAV, HA is an essential factor due to its structure and key role in viral entry into host cells. There are studies indicating the presence of an intrinsic virulence factor in certain HA subtypes of avian strains. For instance, one study demonstrated that the HA molecules of avian strains (H1, H6, H7, H10 and H15) show phenotypes of a similar severe inflammatory type [17].

Among the viral proteins used by IAV to modulate host cell defence responses is the NS1 protein. NS1 has a multitude of functions and is notable at neutralizing IFN response at multiple stages [18]. NS1 is an RNA‐binding protein that inhibits RIG‐1 function. In addition, NS1 can also inhibit host responses to IAV infection by interfering with host mRNA synthesis, processing and trafficking. NS1 may also target IFN‐stimulated genes exhibiting antiviral functions, activator of transcription (STAT) pathway and Janus kinase (JAK)‐signal transducer‐mediated IFN signalling events in order to further antagonize the host antiviral response [19, 20].

The polymerase basic 1 frame 2 (PB1‐F2) is an auxiliary IAV protein derived from the second open reading frame of the PB1 gene. Despite its small size, PB1‐F2 is a critical virulence factor. PB1‐F2 is an ‘immune cell killer’, inducing immune cell apoptosis [21]. Its multifaceted role includes binding to and inhibiting signalling activity mediated by RIG‐1/tripartite motif‐containing protein (TRIM)/mitochondrial antiviral signalling (MAVS), reduces type 1, III IFNs, and downstream induction of host antiviral responses [22]. The pairing of PB1‐F2 and NS1 is a dynamic one as it enables efficiency of IAV replication via effective control of host cellular machineries, and at the same time initiates cytokine storms, and dampening infected individuals' innate immunity [22]. A newly discovered IAV protein, PA‐x, synthesized from the ribosome shift of the viral RNA polymerase mRNA, is another virulence factor affecting the innate immune response. Due to its RNA endonuclease activity, it suppresses expression of host genes [23].

## 3 Human Universal Flu Vaccine

HA head‐specific antibodies (Abs) generate neutralizing responses and form the basis in the production of current influenza vaccines. Unfortunately, the approach to target the HA globular head has been shown to be a double‐edged sword. Although the HA head domain is immunodominant, the epitopes in this region are highly variable. This plasticity allows for increased susceptibility for viral mutation or antigenic drifts, allowing the evasion of the virus from existing immunity [24, 25]. In order to confer protection against new seasonal viruses, annual review and reformulation of influenza vaccines have been carried out to this day [13]. The current strategy utilized for producing seasonal vaccines keeps the world at least a year behind this ever‐changing virus [14]. Hence, the limitations of current influenza vaccines necessitate the need for new or ‘universal’ influenza vaccines, which can confer high efficacy against any potential seasonal, zoonotic or emerging pandemic IAV strain [25]. In line with the National Institute of Allergy and Infectious Diseases (NIAID)'s strategic plan, a universal influenza vaccine (UIV) should fulfil four criteria: It should achieve a 75% effectiveness against symptomatic influenza infection, provide protection against both Group I and Group II influenza viruses, offer long‐lasting protection lasting at least 1 year and be suitable for all age groups. Rapid mutations in influenza viruses contribute to evasion of immunity produced by natural infection or vaccination. Hence, a promising UIV should be able to stimulate both B and T cell responses against various conserved proteins to achieve efficient viral clearance, broad protection, long‐lasting immunity and prevention of reinfection [14].

Currently, multiple vaccination strategies are underway in the quest to develop a broadly protective UIV. Approaches include targeting virus proteins, conserving regions of several virus proteins, developing new platforms and technologies in vaccination and utilizing adjuvants to generate improved immune responses [26]. Target antigens include the conserved stalk domain of HA and conserved epitopes on the HA head, NA, M2e, M1 and NP [25]. In recent years, several potential UIV candidates have advanced to clinical trials (Table 1). In the past decade, the discovery of broadly neutralizing Abs (bnAbs), which are Abs with the ability to neutralize various influenza strains and subtypes, has provided significant insight into the structural and therapeutic design of UIVs. The receptor‐binding site (RBS) of HA and the HA stem are the targets of these bnAbs [38, 39, 40].

**TABLE 1:** List of available influenza vaccine antigens target and its development stage.

Immunogen	Vaccine name	Platform	Adjuvant	Manufacturer	Development stage/Clinical trial ID/Year	Clinical studies	Ref.
HA	PanBlok H7	Recombinant proteins	AS03 or MF59	Vaxine Pty (Australia)	Phase 2 NCT03283319 2017–2018	Induced high cross‐reactivity to antigenically distinct heterologous A(H7N9) viruses	[27]
HA	Quadrivalent VLP (QVLP)	Plant‐derived VLPs	Alhydrogel	Medicago	Phase 3 NCT03301051 2017–2018	Did not meet its primary endpoint of 70% absolute vaccine efficacy against respiratory illness caused by matched strains	[28]
HA	VXA‐A1.1	Adenoviral‐vector based	TLR3 agonist	Vaxart	Phase 2 NCT02918006 2016–2018	Well tolerated and elicited protective immunity against virus shedding	[29]
HA	VAL‐506440	mRNA‐LNP	None	ModernaTX, Inc.	Phase 1 NCT03076385 2015–2018	Well tolerated and induced robust humoral immune response but did not induce significant cellular response	[30]
HA	VAL‐339851	mRNA‐LNP	None	ModernaTX, Inc.	Phase 1 NCT03345043 2016–2018	Well tolerated and induced robust humoral immune response but did not induce significant cellular response	[30]
HA	PF‐07252220	mRNA	None	Pfizer	Phase 1/2 NCT05052697 2021–2023	Trial results expected to be available in the near future.	[31]
HA	FluMos‐v1	Nanoparticle‐based	SAS	NIAID	Phase 1 NCT04896086 2021–2024	Ongoing	[32]
HA	FluMos‐v2	Nanoparticle‐based	SAS	NIAID	Phase 1 NCT04896086 2023–2024	Ongoing	Not published
cHA	cHA‐based LAIV combinations	Genetically modified	AS03A	Icahn School of Medicine at Mount Sinai (USA)	Phase 1 NCT03300050 2017–2019	Induced high anti‐stalk Ab titers and long‐lasting immunity	[33]
Headless HA/HA stem	H1ssF	Ferritin nanoparticle	None	NIAID	Phase 1 NCT03814720 2019–2022	Induced cross‐reactive neutralizing abs against the conserved HA stem of group 1 influenza viruses	[34]
Headless HA/HA stem	EBS‐UFV‐001	Non‐VLP nanoparticle	Aluminium hydroxide and CpG	Emergent BioSolutions	Phase 1 NCT05155319 2021–2024	Ongoing	Not published
Headless HA/HA stem	H2HA‐ferritin	Ferritin nanoparticle‐based vaccine	None	NIAID	Phase 1 NCT03186781 2017–2020	Well tolerated; induced broad neutralizing antibody responses against group 1 influenza viruses	[35]
Headless HA/HA stem	H1ssF‐3928	mRNA‐LNP	None	NIAID	Phase 1 NCT05755620 2023–2024	Ongoing	Not published
M2e and HA stem	Uniflu	Recombinant protein	Flagellin	VA Pharma Limited Liability Company	Phase 1 NCT03789539 2018	Trial results expected to be available in the near future.	[36]
M1, NP, and HA	Multimeric‐001 (M‐001)	Recombinant protein	None	BiondVax Pharmaceuticals	Phase 3 NCT03450915 2018–2020	Did not protect from influenza‐like illness or influenza infection	[37]

HA stalk‐reactive Abs protect the host cell via a mechanism that involves locking the HA trimer protein in a pre‐fusion conformation that prevents conformational changes in the endosome triggered by low pH. As the conformational change is responsible for viral membrane fusion to the host cell membrane, the static HA structure thus prevents the viral genome from being introduced to the host cell [40].

An additional mechanism was also identified in the inhibition of NA protein. HA stalk‐reactive Abs were found to inhibit the enzymatic activity of NA through steric hindrance, which limits NA from accessing sialic acids when adjoining HA. Another study utilizing a microscopy‐based assay further demonstrates the role of HA stalk‐reactive Abs as an obstacle for virus particle egression [24, 41].

Due to the highly conserved nature of the stalk domain of HA, the HA stalk–based approach is the focus of current UIV development concepts and forms the basis of ongoing clinical trials. The HA stalk has a lower mutability compared to the HA globular head. Abs directed against the HA stalk domain are found to be capable of neutralizing heterologous influenza viruses, due to the relative conservation of the HA stalk across divergent subtypes of influenza [42]. However, the proximal stalk domain along with conserved parts of the head domain of HA is immunosubdominant [43]. Therefore, vaccine approaches involving novel constructs of HA and vaccination strategies are necessary to redirect Ab responses towards the conserved HA stalk [44].

## 4 Universal Vaccine Platforms

An ideal UIV should possess the ability to induce heterosubtypic immunity against all influenza A and B viruses while sustaining long‐lasting immune responses. It is also crucial to protect the antigens from rapid degradation and diffusion within the body. To facilitate the successful development of effective UIVs, recent scientific advances have paved the way for novel vaccine development strategies. These strategies encompass innovative vaccine platforms such as nucleic acid–based delivery, viral vectors, recombinant proteins, virus‐like particles (VLPs) and nanoparticles [45, 46]. Various novel platforms have been successfully applied in the development of potential UIVs that have demonstrated vaccine efficacy in clinical trials (Table 1). Another strategy to improve immune responses is through the use of complementary adjuvants in vaccine formulations. Co‐administration of appropriate adjuvants with antigens will enhance the scale and breadth of the body's immune response with a dose‐sparing effect, hence contributing to the vaccine's protective ability against heterologous influenza strains. Various particulate adjuvants for injection and mucosal routes of administration have been evaluated.

VLPs share morphological and structural similarities to the virus, but they do not possess the viral genome. They are obtained through self‐assembly of viral structural proteins carrying antigens. Because of their natural characteristics and their ability to directly activate antigen‐presenting cells, the immune system recognizes VLPs in a manner similar to viruses, inducing both B cell and T cell immune responses [47, 48]. VLPs have been widely used as platform for HA‐, M2e‐ and NA‐based vaccines. The VLP‐induced immunogenicity can be further enhanced by co‐administration with adjuvants such as flagellin, alhydrogel and toll‐like receptor ligands. However, some of the main challenges associated with VLPs are lower stability, difficult upstream and downstream processing, high production costs and appropriate VLP assembly [49].

Nano‐vaccine technology involves encapsulation of antigens into nanoparticles or the displaying antigens on their surface. This approach protects antigens from premature proteolytic degradation, improves stability, possesses good adjuvant properties, facilitates targeted delivery and controls their release, leading to enhanced antigen presentation, and subsequently results in increased vaccine antigenicity and immunogenicity [50, 51]. A study demonstrated that two‐component nanoparticles that co‐display diverse influenza HA trimers induced broadly protective antibody responses to heterologous viruses in mice, ferrets and nonhuman primates (NHPs) [52]. Self‐assembling protein nanoparticle, ferritin, has been successfully employed to induce broadly neutralizing Abs directed against the conserved HA stem in H2‐naive adults [53]. Double‐layered protein nanoparticles fabricated by desolvating M2e or NP into protein nanoparticle cores and crosslinking HA stalks, NA or NP as coating antigens on the core surfaces have been developed [27, 28, 29]. HA stalk and M2e core layered protein nanoparticles were found to protect mice against divergent influenza strains and induce long‐lasting immunity [28]. Results from these studies demonstrate the potential of layered protein nanoparticle nanoplatform as a general vaccine platform. Other nanoparticles that have been used in developing UIVs include liposome, PLGA [30], chitosan [31] and acetalated dextran (Ace‐DEX) [32].

The positive outcomes of mRNA vaccines against SARS‐CoV‐2 in clinical settings have inspired further research and development of mRNA‐based vaccines for various other pathogens [33, 34]. mRNA vaccines offer the potential for fast, scalable and efficient large‐scale production, coupled with the precise design of antigens [35, 36]. This allows in responding promptly and effectively to emerging threats posed by influenza and other pathogens. mRNA vaccines carry out their functions completely within the cytosol avoiding, thereby avoiding the risks associated with nuclear delivery and integration into the genome [37]. A single mRNA can encode multiple antigens, bolstering the immune response against resilient pathogens, viral variants with a single formulation. The mRNA‐based vaccine platform has been explored for multivalent combinations of influenza antigens for induction of broadly protective immunity against a wider range of potential pandemic influenza strains [54, 55]. Another study demonstrated that mRNA vaccines are capable conferring protection against antigenically variable viruses by eliciting the production of Abs targeting multiple antigens at the same time [56]. mRNA vaccines come in two main categories based on its mechanism of actions: conventional mRNA vaccines and self‐amplifying mRNA (SAM) vaccines [57]. SAM vaccines do not use modified nucleosides, while non‐replicating mRNA vaccines can be made with or without the incorporation of various modified nucleosides [58]. A number of mRNA‐based seasonal flu vaccines as well as UIVs are currently in the clinical trials [59, 60]. However, effectively delivering mRNA molecules with precision remains a substantial challenge due to their inherent instability and vulnerability to degradation.

## 5 HA‐Based UIVs

### 5.1 Chimeric HAs (cHAs)

One promising strategy aimed at eliciting Abs against the immunosubdominant HA stalk is the utilization of cHAs. This involves chimerization of HA to encode the HA head from exotic HA head domains from avian subtypes of which humans are naive to such as avian H8 and HA stalk domain of seasonal influenza viruses. As the HA stalk domain is previously encountered, Abs directed towards the conserved stalk will be boosted. By administering a subsequent vaccination with a cHA that has a different exotic head but the same stalk, Abs can be further amplified [61, 62].

A phase 1 clinical trial aiming to evaluate the safety and ability of cHA‐based UIVs to evoke broadly cross‐reactive Abs targeting the HA stalk was recently concluded [62]. The testing was conducted in American adults aged 18–39 years old. In this study, the cHA constructs carrying H8 and H5 head domains grafted onto an H1 stalk were used. The five‐group study consisted of three vaccine regimens and two placebo control group and are as follows; Group 1 received live‐attenuated influenza virus vaccine (LAIV) expressing cH8/1 HA followed by a cH5/1 HA‐expressed ASO3‐adjuvanted inactivated influenza virus vaccine (IIV). Group 2 also received a similar LAIV to group 1, but the IIV boost was without adjuvant. Group 3 acted as a placebo control for Groups 1 and 2. Group 4 received twice ASO3‐adjuvanted IIV. Group 5 received intramuscular PBS and served as a placebo control for Group 4. In terms of safety, all the vaccine regimens had acceptable adverse‐effect profiles on par with other similar approved vaccines. Remarkably high anti‐stalk Ab titres were found to be induced by vaccination with the adjuvanted, inactivated cHA vaccines. This effect was seen even after a singly administered vaccine. Long‐lived antibody titres were observed especially in Group 4. Moreover, antibody responses induced by the vaccines demonstrated Fc‐FcR‐mediated effector functional activity, which is important in protecting anti‐stalk Abs in vivo in animal models. In conclusion, the study found that vaccination with the approach used was safe and able to generate broad and long‐lasting immune responses, hence demonstrating the potential of cHAs as a promising strategy in the development of next‐generation influenza vaccines. Numerous other animal models involving the use of cHAs in the past have also shown the generation of broadly protective Abs [63, 64]. The ongoing development of cHAs consisting of group 2 stalk and IBV mosaic HA (mHA) vaccine presents the possibility of combining all three constructs into a trivalent vaccine capable of protecting against all influenza virus strains [62, 65].

### 5.2 Headless HAs

An alternative strategy that aims to redirect the immune response to the immunosubdominant stalk from the immunodominant head domain of HA is by constructing a ‘headless HA’ whereby the HA head is completely removed [66]. A downside to this approach is the resulting instability of HAs without the head, making it challenging to develop stable constructs as conformation‐dependent epitopes are targeted by the majority of cross‐reactive Abs [67].

In a previous study testing stabilized‐stem (SS) HA from H1 and H3 subtypes fused to ferritin nanoparticles on mice and ferrets, encouraging protective responses were elicited, but concerns were highlighted regarding the appropriateness of these animal models to correctly predict antibody responses in humans [68]. Hence, the research group carried out a new trial using NHPs as test subjects to evaluate the immunogenicity of SS nanoparticle vaccines, of which the results were recently published. The study demonstrated that bnAbs neutralizing diverse H1 and H3 subtypes could be induced in NHPs through immunization with both H1 and H3 stem nanoparticles when administered with the adjuvant AFO3. The resemblance of the monoclonal Abs (mAbs) to human bnAb prototypes in terms of potency and binding specificity was also found to be significant. Despite lacking the immunoglobulin germline VH1‐69 residues, other gene families in NHPs were able to generate bnAbs similar to human bnAbs [66]. An alternative approach involved structurally engineered stable HA stem constructs, termed ‘mini‐HAs’. Mini‐HAs in mice and NHPs were found to elicit Abs that had broadly protective immune responses. This proof‐of‐principle study shows that HA stem mimics design can evoke bnAbs against group 1 IAV [69]. Currently, there are a number of headless HA–based vaccines in the clinical trials at various stages (Table 1).

### 5.3 mHAs

The induction of cross‐reactive Abs against both the HA head and stalk domain would be ideal in a UIV candidate. Distinct antigenic sites on the head domain are mostly the focus of the unequally distributed immune response directed towards the head. By replacing only the major strain‐specific antigenic sites of the head domain with corresponding sequences from exotic, avian influenza virus subtypes, the dominance of these sites can be overcome and redirected to the more conserved epitopes. These mHAs are also thought to elicit and boost cross‐reactive Abs against the immunosubdominant conserved epitopes in the HA head in addition to stalk Abs. Several preclinical mouse studies have demonstrated this unique ability [65, 70]. Like the cHAs, mHAs utilize sequential vaccination strategy and are regarded as a potential method of improving upon the cHAs approach [25, 65].

### 5.4 HA mRNA

The first mRNA vaccines were developed for H10N8 and H7N9 influenza viruses, and it is not only well‐tolerated but also effectively triggered strong antibody‐based immune responses [60]. This study used a lipid nanoparticle (LNP)–formulated mRNA vaccine platform. The mRNA vaccines were composed of chemically altered mRNA molecules that contained the complete, membrane‐bound version of the HA glycoprotein derived from the H10N8 influenza strain or the H7N9 influenza strain. The outcome of the clinical trials was positive, with no vaccine‐related serious adverse events reported. Seroconversion rates were 78.3% for haemagglutination inhibition (HAI) and 87.0% for microneutralization (MN) assays in the case of H10N8 and 96.3% for HAI and 100% for MN in the case of H7N9 [60]. Arevalo et al. developed a nucleoside‐modified mRNA–LNP vaccine encoding HA antigens from all 20 known subtypes of influenza A and B viruses. The vaccine induced high levels of cross‐reactive and subtype‐specific Abs in mice and ferrets, which protected these animals from both closely related and unrelated influenza virus strains [56]. Another nucleoside‐modified mRNA‐LNPs vaccine targeting the conserved HA stalk domain demonstrated induction of broadly reactive antibody responses in mice, which resulted in protection against homologous, heterologous and heterosubtypic influenza viruses [71]. An mRNA vaccine H1ssF‐3928 mRNA‐LNP is currently in phase 1 clinical trial. The vaccine is based on an earlier UIV candidate that has already demonstrated positive results in early clinical trials. Both vaccines employ a distinct section of the flu protein, HA to stimulate a comprehensive immune response against influenza [72].

### 5.5 Computationally Optimized Broadly Reactive Antigens (COBRA) HA

Over the years, various consensus‐based approaches for the HA protein have been designed as a means to overcome influenza vaccines capable of eliciting bnAbs [73, 74, 75]. In short, the COBRA methodology uses multiple rounds of layered consensus building, a process that begins by deducing the phylogenetic tree based on HA amino acid sequences. The final consensus sequences (COBRAs) are then tested on their functional ability to evoke immune responses [76, 77]. Studies utilizing COBRAs representing H1N1, H3N2 and H5N1 influenza subtypes were shown to elicit bnAbs in mice, ferrets an––d NHP studies [76]. Studies utilizing COBRAs representing H1N1, H2N2 and H3N2 influenza subtypes have been shown to induce Abs with HA inhibitory and neutralization activity against historical as well as variant cocirculating strains in mice and ferrets [10, 73, 74, 77]. Despite their superiority in inducing broad immune protection compared to wild‐type HA vaccines, they have not advanced into the clinical setting.

### 5.6 mHA Nanoparticles

A unique vaccine approach involving mosaic nanoparticles uses the ‘immunosubversion’ principle to generate cross‐reactive Abs. The vaccine is developed using a mosaic of heterotypic HA receptor binding domains that are displayed on a single nanoparticle. As a result, the activity of strain‐specific B cells would be inefficient or prevented. Cross‐reactive B cells that target conserved epitopes would then have an advantage in activating more effective Ab responses. A study in mice has shown proof of concept for subverting the usual monotypic immunodominance of B cells [78].

## 6 NA‐Based UIVs

NA is the second major influenza virus surface glycoprotein, playing multiple roles in the viral infection cycle. It is notably known for expediting the release of newly formed virions from infected cells by cleaving the sialic acid residues of the host cell membrane [79]. NA has been the target of licensed small‐molecule inhibitors, such as oseltamivir, peramivir, zanamivir and laninamivir [80, 81]. Several studies have demonstrated that the NA‐based vaccines could induce broadly protective Abs in animal experiments [79, 82, 83]. In addition, studies have also revealed that incorporating the conserved epitope of the NA can significantly contribute to their broad cross‐reactivity [83, 84]. COBRA methodology has also been explored to design NA vaccine to cross‐react with human, swine and avian influenzas viruses of the N1 NA subtype. The vaccine protected mice from mortality and reduced virus titre [83]. There is evidence to support that anti‐NA Abs decline at slower rates when compared to anti‐HA Abs [85]. Furthermore, due to NA's slower rate of antigenic drift over time in comparison to HA, anti‐NA Abs can maintain their effective binding activity for longer durations, resulting in reduced necessity for frequent updates of NA‐based vaccines [86, 87]. Despite the potential of NA as an immunogen in vaccine development, current UIV efforts are mainly focused on HA‐associated immunity [88]. A simple way of improving breadth and efficacy of current seasonal vaccines would be to supplement the vaccine with NA. Vector‐based approaches and a recent approach using consensus NA immunogens are promising ways in utilizing NA as a component in future universal vaccines [89].

## 7 Matrix Protein 2 Ectodomain (M2e)–Based UIVs

The M2 ion channel is a minor influenza virus surface protein, which is responsible for disassembly of the viral core and assembly and budding of the virus. Its extracellular domain (M2e) is a promising candidate for a UIV because it is a highly conserved region across all IAV subtypes [90, 91]. However, the small M2e epitope is poorly immunogenic, therefore relying on design approaches such as displaying it on VLPs or conjugating it to other carriers to improve immunogenicity [92]. A study demonstrated that recombinant LAIV candidates encoding four M2e epitopes induced high levels of M2e‐specific Abs and mediated cross‐protection in a preclinical ferret model [93]. A recombinant anti‐Clec9A monoclonal antibody fused to M2e induced a prolonged anti‐M2e antibody response and a robust anamnestic protective response against H1N1/PR8 virus. Furthermore, Clec9A–M2e vaccination enhanced preexisting anti‐M2e titres resulting from previous flu exposure [94]. Another recombinant vaccine made of four copies of M2e fused within the immunodominant region of the hepatitis B virus core antigen (HBc) elicited high IgG titres and provoked memory T‐cell formation in mice, resulting in reduced virus titres and increased survival in mice [95]. This vaccine has been evaluated in a single‐site, randomized, double‐blind, placebo‐controlled study (NCT03789539), with the trial results expected to be available in the near future (Table 1). M2e remains a challenging target for a universal vaccine as its IgG‐mediated protection is derived from Fc‐mediated Ab functionality and does not offer sterilizing immunity [96]. In a human influenza challenge model, a passively transferred human mAb to M2e managed to decrease the viral load [93]. The use of adjuvants with an M2e‐based vaccine may induce a more sustainable immune response [97]. As the low protective efficacy of M2e‐based vaccines remains a hurdle, the combination of M2e with other influenza antigens or incorporation as a supplement in other vaccines would be more ideal instead of a standalone approach.

## 8 Multiple Protein‐Based UIVs

The NP and M1 internal virus proteins have relatively conserved sequences, which contain many conserved T cell epitopes [98]. NP in particular is conserved across IAV strains and is currently studied as a target of T cell immunity [99]. Following immunization with NP and M1‐based vaccines, a broad‐spectrum T cell response produced. The stimulation of both CD4+ and CD8+ T cell responses contributes to immunity against influenza virus by limiting disease severity and duration. A novel vaccine, the MVA‐NP + M1, a modified vaccinia Ankara (MVA) vector expressing both NP and M1, has been in a Phase 1 study and deemed to be well‐tolerated in adults, albeit the small sample size. However, subsequent phase IIb trials failed to reach their primary endpoints. Vaccitech, the University of Oxford team that developed the vaccine, no longer lists the MVA‐based vaccine as a pipeline project under development on the website [45, 100]. A phase III trial involving Multimeric‐001 (M‐001), a single recombinant protein containing conserved epitopes from the HA, NP and M1 proteins, did not provide protection against influenza‐like illness or influenza infection (NCT03450915) (Table 1). However, studies have demonstrated that that M‐001 can prime for humoral responses to influenzas antigens [101, 102]. In contrast to these findings, a recent study found that when M‐001 used as a priming vaccine before administration of quadrivalent inactivated influenza vaccine (IIV4) induced polyfunctional T cell responses but did not enhance HAI or microneutralization antibody responses (NCT03058692) (Table 1) [103].

Huber et al. designed a peptide‐based vaccine composed of peptides derived from NP, PB1 and M1, which also included a number of T cell epitopes and a B cell epitope consisting of the conserved HA2 and M2e peptides. Vaccination with the conserved B and T cell epitope‐based vaccine reduced viral loads in the lungs and disease severity in mice and ferrets [104]. Another peptide‐based vaccine, Vacc‐FLU, consisting of one Me3 peptide induced B cell responses, and three peptides representing the M2, NP and a mixed M2 and NP elicited T cell responses in a murine model. The vaccine protected the mice from severe disease symptoms and provided partial protection [105]. Tutykhina et al. designed a recombinant human adenovirus carrying the consensus sequences of M2 and NP proteins of IAV enriched with B and T cell epitopes. The vaccine elicited strong and long lasting CD8 and CD4 T cell responses [106].

A nucleoside‐modified mRNA‐LNP influenza vaccine targeting several conserved antigens (HA stalk, NA, M2 and NP) induced potent antigen‐specific Abs in mice [55]. The vaccines provided effective immunity, even when challenged with a pandemic H1N1 virus at a dosage 500 times greater than the median lethal dose, following just a single immunization. The vaccine coffered protection against infection with seasonal influenza virus, heterologous challenge within the H1N1 subtype and heterosubtypic challenge with viruses bearing avian glycoproteins. Notably, delivering multiple antigens in combination did not result in significant variations in the strength of humoral immune responses when compared to the delivery of a single antigen on its own [55]. van de Ven et al. [107] developed T cell inducing nucleoside‐modified mRNA vaccine that encodes the conserved internal proteins NP, M1 and PB1 of H1N1. The vaccine was evaluated as a prime‐boost regimen in naïve ferrets and as a booster in influenza‐experienced ferrets. A single dose of the vaccine elicited cellular responses against NP but not to M1 and PB1. However, the immune responses against all three antigens NP, M1 and PB1 were stronger and broader in H1N1‐primed ferrets. Moreover, the booster vaccination increased protection against heterosubtypic infection caused by the potential pandemic H7N9 influenza virus in ferrets with prior exposure. Another study found that simultaneous administration of mRNA vaccines encoding different antigens, including HA and NA, showed increased efficacy, suggesting that a combination of these antigens would give a broadly protective vaccine [108].

## 9 Advantages of a UIV

The NIAID and the WHO has prioritized the concept of a broadly protective UIV targeting conserved epitopes, as a research area [109]. Success in developing a UIV would undoubtedly be a worthy countermeasure against the ever‐evolving influenza virus. The current ongoing threat posed by influenza viruses to public health through causing significant morbidity, mortality and economic burden would make the payoff of UIV even more extensive in terms of public health benefits. In the United States, an amount of $6.6 billion was spent on distributing influenza vaccines during the 2015/16 influenza season [110]. As current vaccines require updating and re‐administration annually, billions of dollars are needed every year. Moreover, cumulative costs of obtaining seasonal influenza vaccines could be estimated to cost at least $3500/person/lifetime. A broadly protective UIV that may only require two to three times administration in a lifetime would therefore cost significantly less than the existing standard of care [110, 111]. The reduced frequency of doses from getting vaccinated by a UIV would invariably improve vaccine uptake and coverage due to solving issues that arise from annual revaccination such as time, cost and needle aversion [111]. Low‐ and middle‐income countries, in particular, would reap the benefits of a less costly UIV independent of a vaccine match [112]. A UIV would also be considerably advantageous with regard to enhancing pandemic preparedness. As the process of producing new matched vaccines to protect against pandemic strains takes approximately 6 months, the population remains vulnerable during this long period of time. Regardless of vaccine coverage, the threat of any emerging pandemic strain can be completely eradicated [110].

## 10 Challenges, Limitations and Important Considerations in the Development of UIVs

Significant progress has been made in the development of effective UIVs, with several vaccines demonstrating broad and long‐lasting protective immunity, especially in clinical trials. However, there are a number of challenges and limitations that are need to be addressed in order to improve their efficacy. One of the challenges is the absence of a standardized preclinical evaluation process and the lack of a definitive animal model that accurately mimics human infection. Host factors such as pre‐existing immunity, gender, age and underlying chronic conditions may impact vaccine efficacy. Achieving long‐term protection, ideally for at least 1 year, also presents a considerable challenge for UIVs. This goal is likely to be accomplished through immunization schedules, formulations, adjuvants and dosages.

Several significant challenges remain when it comes to the possibility of generating broad protection through immunization with highly novel influenza strains in humans. This is due to a majority of influenza‐activated B cells originate from memory cells, and most of these memory B cells have variable genes that have undergone somatic mutations [43, 113]. The current efforts in developing a UIV centre around the two conserved epitopes on the influenza HA: the ‘stem’ and the RBS on the ‘head’ [39]. Existing immune memory against the head epitopes of HA takes precedence, preventing the development of a broadly effective immune response to the HA stalk when individuals are revaccinated with similar strains [43]. Labombarde et al. examine whether broadly reactive influenza Abs, which may also bind self‐antigens, play a role in autoimmunity. They demonstrate that the induction of these Abs also promotes the development of autoreactive Abs in both humans and mice. The transfer of broadly reactive mAbs exacerbates autoimmunity in the presence of inflammation or genetic susceptibility [114]. Overall, these data imply that mechanisms for self‐tolerance act to restrict the occurrence of broadly reactive influenza Abs, which have the potential to worsen the disease when combined with additional risk factors.

Currently, the correlates of protection used for seasonal influenza vaccines is based on the HAI and single radial haemolysis (SRH) assays. Regulatory agencies have long held these serological assays as a correlate of protection for licensing approval of inactivated influenza vaccines [115]. The HAI assay is based on the principle of HA binding to sialic acids on the surface of erythrocytes and measures the inhibition of erythrocyte agglutination mediated by Abs. Thus, the HAI assay only detects anti‐HA head Abs as the RBS is located at the HA head domain. Abs elicited in UIV strategies that target the conserved epitopes of the HA stalk, M2e, NA, NP or M1 would be undetectable by the HAI assay. HA stalk–reactive Abs have been described to have multifunctional protection mechanisms and can induce Fc‐dependent effector functions such as antibody‐dependent cellular cytotoxicity (ADCC) or phagocytosis (ADCP) [116, 117]. Furthermore, the SRH assay is also not applicable in measuring next‐generation vaccination strategies such as those targeting M1 or NP as the assay is unable to recognize mucosal immunity or cell‐mediated immunity [115]. Seasonal vaccines require evidence of efficacy in order to be granted licensure. With the lack of a well‐established correlate of protection to demonstrate clinical efficacy, UIVs face a major obstacle with regard to obtaining regulatory approval [112, 118]. Several novel correlates of protection such as HA stalk–specific Abs and nasal IgA have been identified and undergoing investigations in the hope of facilitating UIV development [119].

There is potential, albeit little for broadly protective UIV to influence the evolution of influenza viruses. This is because the vaccines may introduce new selection pressures from redirecting immunity towards non‐target proteins of natural immunity. In the event of a pandemic, high vaccine utilization worldwide could also strengthen selection [110, 120]. Although the risk of escape mutants from UIV targeting conserved regions is low, observations on influenza evolution have only been confined to experimental settings, which do not always reflect real‐world scenarios [120]. A study by Park et al. has raised concerns regarding the possible emergence of escape mutation(s) in the HA stalk under immune selective pressure [121].

UIV efficacy may be affected depending on prior immunity. Vaccine efficacy may be reduced if a population has a biased prior immunity [109]. Due to factors such as original antigenic sin (OAS) and preexisting host immunity that is influenced by individual age, sex, exposure history or immune status, UIV strategies may need to be specifically tailored to different populations in order to be fully efficacious [110, 116, 122].

Any issues concerning immunity and risks of UIVs could be addressed early using human challenge models [112]. Testing UIVs in challenge studies would rapidly ascertain their efficacy and allow them to be directly compared with current influenza vaccines with both matched or mismatched seasonal virus strains [110]. Furthermore, there is gaining acceptance regarding human challenge trials by regulatory agencies for UIVs lacking traditional correlates of protection [115].

The vast economic cost of developing a broadly protective UIV, coupled with the constant revenue stream earned by the vaccine industry from annual re‐administration of influenza vaccines, may curb the incentive to develop UIVs. Nevertheless, the incentive for manufacturers to obtain an unrivalled market position through UIVs remains and continues to drive emerging growth in UIV strategies [110, 123].

## 11 Conclusion

In the last decade, immense progress in developing strategies to produce a UIV has been made. Novel platforms with innovative technologies that utilize VLPs, nanoparticles and mRNA have been successfully applied in the development of UIVs and have demonstrated their effectiveness in both preclinical and clinical stages. In addition, the use of adjuvants has been shown to enhance antigen immunogenicity and vaccine efficacy. The advances in novel vaccine platform technologies have and will continue to pave the way for breakthroughs in UIV design and development. Various viral targets, particularly the conserved regions in the HA, NA, M2e and NP, have been utilized in the development of UIVs. Targeting the conserved regions of specific antigens as well as multiple proteins has shown to induce broad protection against a wide range of influenza viruses. In order to elicit a broad protection against different antigenic subtypes and lineages of influenza A and B viruses, ideally, the vaccine should be able to stimulate both humoral and cellular immune responses. Despite existing numerous challenges and considerations in UIV development, cross‐collaborative research and results from ongoing trials will surmount these issues. In the coming few years, as more evidence on UIVs becomes available, the goal in finally achieving a UIV will not be so far‐fetched.

## Author Contributions

**Caryn Myn Li Lim:** Writing – original draft. **Thamil Vaani Komarasamy:** Writing – review and editing. **Nur Amelia Azreen Binti Adnan:** Writing – review and editing. **Ammu Kutty Radakrishnan:** Writing – review and editing. **Vinod R. M. T. Balasubramaniam:** Conceptualization; Supervision; Writing – review and editing.

## Conflicts of Interest

The authors declare no conflicts of interest.

### Peer Review

The peer review history for this article is available at https://www.webofscience.com/api/gateway/wos/peer‐review/10.1111/irv.13276.

## Data Availability

Data are included in article or are referenced in the article.
